# Patient satisfaction after implementation of person-centred handover in oncological inpatient care – A cross-sectional study

**DOI:** 10.1371/journal.pone.0175397

**Published:** 2017-04-06

**Authors:** Anna Kullberg, Lena Sharp, Hemming Johansson, Yvonne Brandberg, Mia Bergenmar

**Affiliations:** 1 Department of Oncology-Pathology, Karolinska Institutet, Stockholm, Sweden; 2 Department of Learning, Informatics, Management and Ethics, Karolinska Institutet, Stockholm, Sweden; 3 Regional Cancer Centre, Stockholm-Gotland, Stockholm, Sweden; 4 Department of Oncology, Karolinska University Hospital, Stockholm, Sweden; 5 Center for Digestive Diseases, Karolinska Univserity Hospital, Stockholm, Sweden; Universite de Bretagne Occidentale, FRANCE

## Abstract

Effective nurse shift-to-shift handover is a prerequisite for high-quality inpatient care. Combining person-centeredness with the need for improved handover rituals, we introduced and evaluated person-centered handover (PCH) in an oncological inpatient setting. PCH is the shift-to-shift nursing report performed together with the patient according to a set structure focused on patient participation, relevant clinical information, and patient safety. Non-verbal handover, standard at the department, is conducted via the electronic health record, in absence of the patient, and without a set structure. The aim of the study was to compare person-centered handover with non-verbal handover in an oncological inpatient setting with regard to patient satisfaction. A cross-sectional design featuring two points of measurement at one intervention ward and two control wards was applied. The EORTC IN-PATSAT32 questionnaire was used for measuring patient satisfaction. Baseline measurements were taken during the spring of 2014, when all three wards used a non-verbal handover model, and included responses from 116 patients. Follow-up measurements (comparing PCH and non-verbal handover) involved 209 patients and were on-going from September 2014 to May 2015. After the introduction of PCH, one change in patient satisfaction was detected regarding the subscale measuring exchange of information between caregivers. Patients from the intervention ward scored statistically higher after the implementation of PCH when compared to the control wards (p = .0058). The difference remained after a multivariate regression analysis controlling for clinical variables. In conclusion, PCH is feasible in oncological inpatient care but does not seem to affect patient satisfaction.

## Introduction

During the last decade, patient satisfaction has been increasingly used as a measurement of quality and performance for health care organizations [1]. Patients’ perception of involvement and information is of vital importance for patient satisfaction [2]. In addition, Swedish legislation puts clear demands on health care providers to involve and inform patients in all areas of care delivery. One dimension of patient satisfaction is information exchange, which is a prerequisite for high-quality health care [3]. As opposed to information transfer, *information exchange* is a two-way dialogue allowing for mutual learning and understanding [4]. It occurs both between professionals and between professionals and patients, and it requires active participation from all parties involved. Insufficient communication can cause misinformation and have serious consequences for participation [5]. Information exchange and/or provision is commonly included in patient satisfaction surveys and considered to be a vital part of patients’ hospital experiences [6]. Satisfaction with information as well as satisfaction with involvement have been revealed as weak points by patient surveys in Sweden [7]. In addition, recent data from the Commonwealth Fund comparing 11 developed countries reveal that Sweden was ranked second last on the indicator “Quality Care”[8]. Patient-centred care, defined as “care delivered with the patient’s needs and preferences in mind”, was one of four categories forming the indicator “Quality Care”.

Person-centred care (PCC) lacks a universally accepted definition, but core elements include establishing a partnership between the patient and the health care professional, whereby the patient is empowered to play an active role, as well as the staff taking the patient’s capabilities, rights and personal preferences into account [9]. PCC includes involving patients and/or family members in the planning and deliverance of health care, with joint goals and strategies [10]. Interventions in care promoting PCC have shown positive effects on, for example, patient satisfaction [11, 12]. The link between patient satisfaction and nursing care has been emphasized and considered to be stronger than, for example, the link between patient satisfaction and physicians’ health care delivery [13].

Striving toward further patient involvement and more effective ways of communication is important in health care. Nursing handovers between shifts have been described as a critical process where misinformation is common, thus increasing the risk of errors in care and impairing continuity [14]. In oncological settings, effective handovers are of utmost importance where the combination of high-risk treatments and critically ill patients could give rise to serious adverse events. In a Danish study, the specific needs of cancer patients in handovers were investigated. The authors found that one is six patients had unmet information- and coordination needs [15].

Traditionally, nurse-to-nurse reports are conducted at the nurses’ station, excluding patients. Nursing handover, however, could be an opportunity to involve patients and family members in information exchange, allowing them to play an active role as partners in care. The concept of “bedside handover” is not new and has been tested and evaluated with various outcomes and in different settings. As the name implies, bedside handover is the shift-to-shift report between nursing staff at the bedside, allowing for, but not necessarily including, patient involvement. Previous studies examining variations of handover at the bedside are often of a qualitative descriptive character [16–19], investigate nurses’ perspectives [20] and have used pre- and/post-implementation designs without control groups [21, 22].

A recent Cochrane review investigated which nursing handover styles are associated with improved patient safety outcomes and nurse processes [23]. No analyses could be undertaken, however, because the authors failed to find any randomized studies. The authors call for high-quality studies to determine the most effective nursing handover models. A more inclusive review compiled nine original papers and found a direct correlation between bedside handover and increased patient and nurse satisfaction, but not specifically in cancer care [24]. There is, however, a growing body of evidence supporting the involvement of patients in nursing handover. Sand-Jecklin *et al*. (2014) conducted a large quasi-experiment evaluating a blend of recorded and bedside handovers with regard to patient and nursing satisfaction, nurse overtime and patient safety outcomes. They found improvement in patient safety and nurse and patient satisfaction but also experienced drawbacks with the implementation process [22]. A multi-centre, prospective mixed-methods study is currently planned in Belgium to evaluate bedside handover in comparison with handover styles not including patient involvement [25], providing an example of current ambitions to deliver high-quality studies in this field of research.

Combining aspects of PCC and the need for improved handover rituals, we introduced and evaluated person-centred handover (PCH) in an oncological inpatient setting: an intervention further described below.

## Aim

The aim of the study was to compare person-centred handover (PCH) with non-verbal handover in an oncological inpatient setting with regard to patient satisfaction.

## Patients and methods

**Fig 1:**
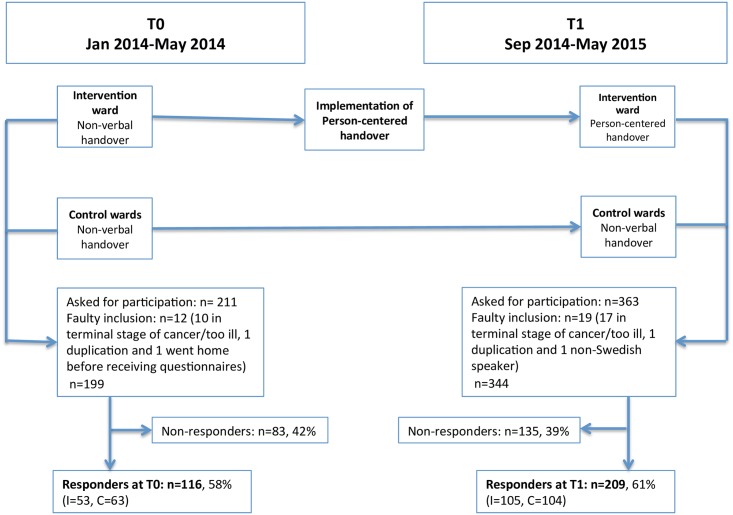
Inclusion scheme and study design from baseline to T1.

The study had a cross-sectional design with two points of measurement, and was conducted at the Department of Oncology, Karolinska University Hospital, Stockholm. Three out of four oncological inpatient wards were included. One served as an intervention ward and the other two as control wards. Baseline data (T0) were collected at all wards from February to May 2014. Thereafter, the intervention, described below, was introduced at the intervention ward, after staff education and training. Data were collected from all three wards from September 2014 to May 2015, following the introduction of the intervention (T1). The design of the study is displayed in Fig 1.

### The setting

The inpatient wards at the Department of Oncology, Karolinska University Hospital provide oncological care for adult patients with cancer. The three wards have different specializations: control ward 1 (C1) cares for patients with gynaecological or breast cancer, control ward 2 (C2) for patients with gastro-intestinal or urological cancer and the intervention ward for patients with head/neck or lung cancer. Patients are in curative and/or adjuvant phase or in palliative stage and undergo radio-, target- and/or chemotherapy. Admissions are either acute or planned. Each registered nurse (RN) is responsible for 4–6 patients during his or her shift. Nurse assistants (NA) provide care for 6–8 patients each. All 18-bed wards have the same staff ratio, the same median length of stay (approximately five days) and a similar nursing workload. The number of admissions per year is approximately 4,200 in total.

### The intervention

Person-centred handover (PCH) concerns the shift-to-shift report between nursing staff together with the patient. PCH is performed according to a set structure with focus on relevant clinical information, including patient safety issues (ID, fall risk, medications and safety concerns raised by the patient). The PCH sessions are led by the morning shift nurse following a checklist (Table 1). The Australian standard operating protocol (SOP)(18) was used, combined with SBAR (Situation-Background-Assessment-Recommendation)[26] for adaption to the local context. Our model was named *person-centred handover* because of its focus on patient involvement and not only a handover conducted at bedside. Prior to the PCH, patients and present family members were asked to take an active part in the handover process and were encouraged to discuss information and to ask questions. The nursing staff work 8-hour shifts, thus there are three shift changes per day. PCH, however, was performed solely between the morning and evening shifts, at the patient’s bedside. PCH was implemented at the intervention ward, before starting the data collection at T1.

**Table 1:** The Person-Centred Handover (PCH) model, description of the five components.

Components	Description
1 Preparation (At nurses’ station)	The nurse coordinator on the morning shift allocates patients and provides a printed list for each member of the evening shift, including patient name, ID, diagnosis for all patients admitted at the ward. The oncoming evening RNs read the admission notes in the electronic health record (EHR). The NA from the morning shift prepare the patient and, if present, family members (according to the patients preferences) that PCH will start shortly
2 Introduction (At bedside)	The RN from the morning shift leads the PCH process (PCH leader) and start with introducing the patient, family members and oncoming staff (RN and NA). The patient (and if relevant, family members) are invited to raise any immediate concerns and/or questions.
3 Information exchange (At bedside)	After initial questions have been dealt with the PCH leader goes through the PCH checklist and discusses the planned care for the day and next coming days. The dialogue is based on SBAR (Situation, Background, Assessment, Recommendation) Medical jargon is avoided The information exchange gives oncoming staff the opportunity to gain 1st hand information from the patients, family members and colleagues. At the end of the PCH procedure, the oncoming RN summarizes and concludes the information exchange and confirms with the patient, the family members and colleagues.
4 Patient involvement (At bedside)	The PCH team leader ensures that the handover process includes repeated opportunities for patients and, if present, family members to express preferences and opinions, seek clarification, ask questions and to be actively involved in any decision related to their care.
5 Safety check (At bedside)	The PCH leader performs a safety check involving the patient, family members and colleagues. The safety check includes; ○ Check that the patient have a correct ID wrist band ○ Fall risk; check fall risk factors, assessment, preventive actions and confirm with the patient ○ Medications ▪ Check that ongoing infusions are correct (in line with prescriptions, infusion time?) ▪ Check any changes in medications (according to EHR) and confirm with the patient and colleagues ▪ Check if the patient or family member have any questions/concerns related to medications ○ Ask the patient and/or family members if they have any safety concerns or have noticed anything divergently

### Non-verbal handover, standard care

On the two control wards (ward C1 and ward C2), the nursing staff continued with the non-verbal handover that was the standard procedure on all three wards prior to the study. At the beginning of each shift, the oncoming nurse spends around 60 minutes reading up on his or her patients from the electronic health records (EHR) in a nursing office, before commencing the shift work and meeting the patients. There is no set structure for which information should be retrieved and how to sift and prioritize. The non-verbal handover was often followed by a short oral information exchange between the off going and the oncoming RNs. This was also performed in isolation from the patients. Non-verbal handover has been described thoroughly in another paper [27].

### Patients

Inclusion criteria were ≥18 years, discharged after a ≥3-day stay, not previously participated in the present study, and having received study information (orally and written) at time of discharge. All patients discharged from the wards during the study periods (T0: January-May 2014; and T1: September 2014-May 2015) were screened by the ward’s nurse-coordinator for participation in the study. Patients who could not speak Swedish or who were in a terminal stage of cancer were excluded.

### Procedures

Further information about the study, the questionnaire and a prepaid return envelope were handed to patients who were interested in participation. They were asked to complete the questionnaires at home and return them by regular mail, using the enclosed prepaid envelope. Each document was coded, allowing the study coordinators to send reminders. A returned questionnaire was regarded as consent to participate. Upon receiving patients’ responses, the study coordinator made a notification in the patient’s EHR to avoid duplications in inclusion.

In cases where no response was received within one week of discharge, a reminder was sent by mail. Patients who were unwilling to participate could avoid the reminder by returning the questionnaires uncompleted.

### Questionnaire

Patient satisfaction was measured by the European Organization for Research and Treatment of Cancer (EORTC) IN-PATSAT32 questionnaire covering the subscales of doctors’ and nurses' technical skills (DTS: 3 items and NTS: 3 items), interpersonal skills (DIS: 3 items and NIS: 3 items), information provision (DIP: 3 items and NIP: 3 items) and availability (DAV: 2 items and NAV: 2 items); other hospital staff's interpersonal skills and information provision (OTH: 3 items), exchange of information (EXE: 1 item) and waiting time (WAI: 2 items); hospital accessibility (ACC: 2 items), comfort (COM: 1 item) and general satisfaction (GEN: 1 item). The EORTC IN-PATSAT32 was specifically developed for hospitalized patients with cancer. Testing has demonstrated excellent internal consistency, convergent validity and high reliability [28].

### Collection of clinical data

The following data were registered from the patients' EHR: gender, age, length of stay, cohabitation, reason for admission, and treatment intention. Data from EHR were registered for all patients who fulfilled inclusion criteria and returned the questionnaires. The patients themselves reported their educational level in the questionnaire.

### Statistical methods

Linear regression models were used to analyse patient satisfaction in the comparison between the intervention ward and the control wards. The results are presented as mean differences, together with 95% confidence intervals. Ordinal responses were tested using the Mann-Withney test and binary responses by a Chi-square test. The effect of the intervention was estimated on its own as well as together with the following covariates: age, gender, treatment intention and educational level.

### Determination of sample size

The mean value and standard deviation (SD) for the subscale “exchange of information between caregivers” (EXE) were 65 and 25 respectively in the large international cross-cultural study in which the psychometric characteristics of the questionnaire EORTC IN-PATSAT32 were assessed (28). The effect of the intervention was assessed at T1. Assuming a mean of 65 and a standard deviation of 25 in the control group for the subscale “exchange of information between caregivers”, a sample of 200 patients (50 recruited from each of the control wards and 100 from the intervention ward) would have a power (1-β) of 80% to detect a true mean difference of 10 units at T1 between patients from the intervention ward and from the control wards using a significance level (α) of 5%. With this sample size, the expected length of a 95% confidence interval for the mean difference would be about ± 7 units. A total of 100 responding patients were needed for the comparison of patients’ characteristics and to identify possible confounding background factors at baseline (T0). The total sample was thus estimated to 300 patients.

The study was approved by the Regional Ethical Review Board, Stockholm (2013/1378-31/2).

## Results

### Patient characteristics

Out of 574 patients fulfilling the inclusion criteria and asked to participate at the three wards during the two study periods, 325 (57%) decided to participate in this study. At baseline (T0), 116 (58.3%) patients participated in the study, and 209 (60.8%) at T1. Fig 1 displays the inclusion schemes for T0 and T1. Clinical variables and background characteristics for responders at both points of measurement are presented in Table 2. Responders from ward C1 were younger than on the other wards at both points of measurements. There were also more female responders from ward C1 at both T0 and T1, reflecting the focus on gynaecological and breast cancer. At T1, the control wards had a larger proportion of responders who were admitted acute than the intervention ward, probably owing to the differing diagnoses.

**Table 2:** Clinical variables for all responding patients from the different wards at T0 and T1 respectively.

	T0				T1			
	C1 n(%)	C2 n(%)	I n(%)	p	C1 n(%)	C2 n(%)	I n(%)	p
Sex								
Female	24(86)	18(53)	34(64)		36(72)	24(45)	44(42)	
Male	3(11)	16(47)	18(34)	.011	14(28)	29(55)	61(58)	.002
Missing	1(4)	0	1(2)		0	1(2)	0	
Age, mean [Sd]	60[15.4]	70[10.0]	65[12.4]	.008	61[14.8]	64[11.5]	67[8.9]	.0084
Co-habitant								
Yes	17(61)	23(67.7)	37(69.8)		30(60)	45(83.3)	77(73.3)	
No	8(28.5)	11(32.4)	13(24.5)	.778	20(40)	8(14.9)	27(25.7)	
Unknown	3(11)	0	3(69)		0	1(2)	1(1)	.016
Education								
Compulsory school ≤9 years	4(14.3)	12(35.3)	10(19.6)		12(25.0)	12(22.6)	25(24.0)	
Upper secondary school, 12 years	11(39.3)	10(29.4)	15(29.4)		7(14.6)	8(15.0)	32(30.1)	
University >12 years	13(46.4)	12(35.3)	26(50.1)	.269	29(60.4)	33(62.3)	47(45.2)	.082
Treatment intention								
Palliative	11(40.1)	26(76.5)	28(57.1)		33(67.4)	36(69.2)	46(43.4)	
Curative	16(59.3)	8(23.5)	21(42.9)	.018	16(32.7)	16(30.8)	59(56.2)	.002
Admission								
Acute	19(70.4)	26(76.5)	36(69.3)		45(90.0)	47(88.7)	64(60.1)	
Planned	8(29.6)	8(23.5)	16(30.1)	.755	5(10.0)	6(11.3)	41(39.0)	.000
Length of stay, mean[Sd]	6[3.1]	7[3.7]	7[3.4]	.160	7[4.7]	7[4.1]	7[4.2]	.10

### Patient satisfaction

**Fig 2:**
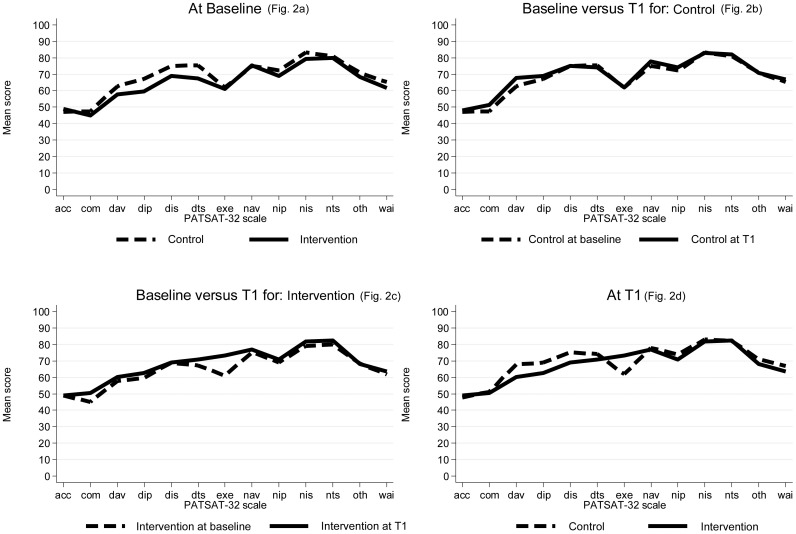
Mean scores of the EORTC INPATSAT-32. (A) shows the mean scores for the intervention ward and the compiled control wards at baseline. (B) shows the mean scores for the compiled control wards at baseline and T1. (C) shows the mean scores for the intervention ward at baseline and T1. (D) shows the mean scores for the intervention ward and the compiled control wards at T1. IN-PATSAT32 scales: ACC = access, COM = comfort, DAV = doctors’ availability, DIP = doctors’ information provision, DIS = doctors’ interpersonal skills, DTS = doctors’ technical skills, EXE = exchange of information between caregivers, GEN = overall quality rating, NAV = nurses’ availability, NIP = nurses’ information provision, NIS = nurses’ interpersonal skills, NTS = nurses’ technical skills, OTH = other personal interpersonal skills and information provision, WAI = waiting time.

At baseline, no significant differences between the three wards were found in any mean scale scores of the EORTC IN-PATSAT32, as shown in Table 3. In Fig 2A, the mean scale scores are displayed for the intervention and the control wards, showing an almost identical pattern. At T1, after the introduction of PCH (Fig 2D), patients from the intervention ward scored statistically significantly higher on the subscale measuring exchange of information between caregivers (p = 0.0058) when compared to the compiled control wards. No other differences in patient satisfaction between the wards were found. A comparison between T0 and T1 within the control group indicated no changes over time (Fig 2B). The corresponding analysis for the intervention group (Fig 2C) revealed EXE to be the only deviant subscale when comparing T0 and T1. Overall, general satisfaction and satisfaction with nurses were high, while access and comfort were consistently lower.

**Table 3:** Mean scale scores for EORTC IN-PATSAT32 over wards and T0 and T1 respectively.

	T0				T1			
EORTC IN-PATSAT32 scales	C1	C2	I	p*	C1	C2	I	p*
ACC [Sd]	42 [22.2]	52 [23.7]	49 [28.0]	0.3082	45[26.1]	50[27.1]	49[26.7]	0.6287
COM[Sd]	53[28.9]	43[36.1]	45[35.7]	0.5219	49[31.7]	54[31.2]	51[29.8]	0.6729
DAV[Sd]	64[28.5]	62[22.7]	59[29.1]	0.6096	71[24.9]	65[24.5]	60[27.7]	0.0541
DIP[Sd]	70[20.2]	65[25.0]	60[30.2]	0.2546	66[29.3]	72[25.1]	63[27.9]	0.1543
DIS[Sd]	74[24.1]	76[19.6]	69[26.7]	0.3743	76[27.0]	75[21.9]	69[24.8]	0.2141
DTS[Sd]	75[18.7]	76[20.6]	67[26.0]	0.1839	72[23.7]	76[21.6]	71[22.0]	0.4124
EXE[Sd]	65[24.3]	60[23.8]	61[28.0]	0.7239	63[26.4]	61[26.7]	73[23.7]	0.0058
GEN[Sd]	80[18.5]	79[23.4]	74[23.4]	0.3251	79[22.2]	80[19.8]	78[22.1]	0.8151
NAV[Sd]	75[24.0]	75[21.1]	75[23.2]	0.9983	75[23.1]	81[19.1]	77[21.6]	0.3628
NIP[Sd]	75[17.3]	70[23.1]	69[26.1]	0.4973	72[25.2]	76[21.1]	71[24.1]	0.3962
NIS[Sd]	83[20.2]	83[22.5]	79[25.1]	0.6430	82[20.9]	84[19.1]	82[17.7]	0.7355
NTS[Sd]	83[22.2]	79[20.6]	80[21.4]	0.8031	80[23.4]	84[17.9]	82[18.3]	0.6562
OTH[Sd]	74[20.4]	68[19.9]	68[23.3]	0.5296	71[20.2]	71[20.6]	68[24.5]	0.6581
WAI[Sd]	69[19.7]	62[24.1]	62[27.5]	0.4371	64[25.7]	70[24.1]	64[27.4]	0.3200

IN-PATSAT32 scales: ACC = access, COM = comfort, DAV = doctors’ availability, DIP = doctors’ information provision, DIS = doctors’ interpersonal skills, DTS = doctors’ technical skills, EXE = exchange of information between caregivers, GEN = overall quality rating, NAV = nurses’ availability, NIP = nurses’ information provision, NIS = nurses’ interpersonal skills, NTS = nurses’ technical skills, OTH = other personal interpersonal skills and information provision, WAI = waiting time

*p-values correspond to F-tests in the linear regression model.

**Fig 3:**
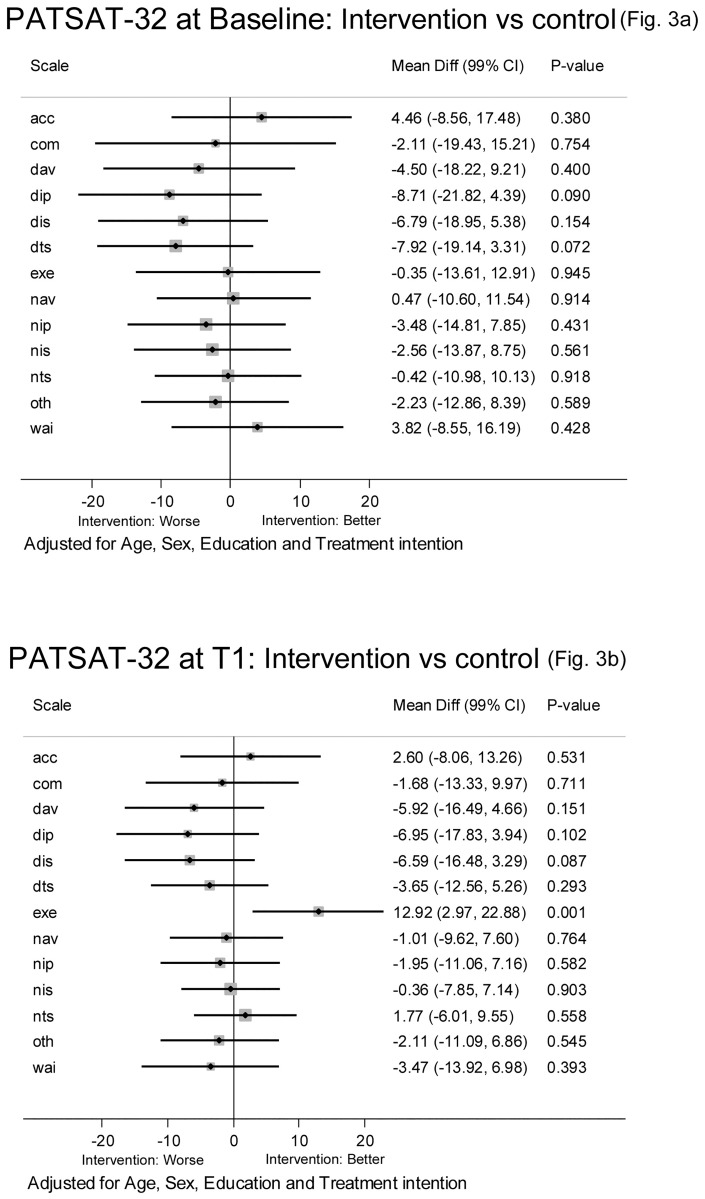
(A) shows the EORTC INPATSAT-32 scores at baseline adjusted for age, sex, education and treatment intention in a multivariate regression analysis. (B) shows the EORTC INPATSAT-32 scores at T1 adjusted for age, sex, education and treatment intention in a multivariate regression analysis.

In a multivariate regression analysis controlling for age, gender, educational level and treatment intention, no differences between the intervention ward and the control wards were found at T0 (Fig 3A). At T1, the same comparison between the groups demonstrated that the difference between the intervention ward and the compiled control wards remained regarding the scale EXE when controlling for clinical variables (Fig 3B).

## Discussion

In this study, PCH was compared with non-verbal handover with regard to patient satisfaction. The results demonstrated no significant differences in patient satisfaction between the intervention ward and the control wards following the introduction of PCH. The single exception regarded how patients rated exchange of information between health care providers, whereby patients from the intervention ward scored higher after implementing PCH. This is, to our knowledge, the only study using a comparison group to contrast non-verbal handover with PCH or similar handover styles.

Patient satisfaction is multidimensional and affected by many variables. The participants in this study had cancer and received inpatient care. Their time at the wards was probably intense, with daily treatments and examinations. An intervention to match those experiences and changing their satisfaction with care has to be powerful. PCH directly affected the patients for around 5 minutes per day. A study using structured observations was performed from January to March 2015 in order to investigate compliance to the PCH checklist among the staff. A total of 21 handovers were observed according to a protocol. No member of staff or any patient was observed more than once. Deficits were found in compliance, primarily regarding the safety check, but every observed handover included an updated enquiry of the patient’s current status and planning for the next 24 hours together with the patient. The handovers took 5.8 minutes on average, ranging from 2 to 10 minutes (unpublished results). Results from the observations were presented to and discussed with staff at the wards to improve compliance. These observations indicate that compliance with the set structure varied and might have affected the results of the present study.

As exchange of information between health care providers was the only subscale that differed significantly between the control and intervention groups in our study, it is relevant to consider what might have caused the improvement. Firstly, during PCH patients play an active role in planning their care and get to participate in nursing staff’s shift change. It is therefore reasonable to expect patients in the intervention group to rate health care providers’ exchange of information higher, as they are actually involved. This is in contrast to patients in the control group who were not given the chance to take part in the communication between members of staff at any time during their hospital stay. Secondly, factors could be present without our knowledge, as there was no means of randomly assigning patients or staff to either the intervention group or the control group. It is, for example, possible that the enhanced focus on communication, safety and handover issues in the intervention ward during the study period spilled over and affected nurses’ information exchange not directly relating to PCH. Thirdly, PCH enhances handover structure, facilitating care continuity for RNs during the evening shift. If patients note this change, it could affect their perception of nurse-to-nurse information exchange [12]. It is also possible that the patients treated at the intervention ward recognized this process to a greater extent, as they were aware of the PCH study. In the intervention group, there was a statistically significantly higher proportion of patients whose admission to the ward was planned, compared to the control group at T1. In general, these patients were in a better physical condition at admission than those admitted acutely. Therefore, they might be able to take a more active part in the handovers. On the other hand, acutely admitted patients’ health status usually improve during the hospital stay, while patients planned for treatment often decline or suffer from side effects while admitted. Admission type as a possible confounder is therefore unclear.

We thought that the information subscales, especially the nurses’ information provision, would follow the same pattern as the subscale regarding information exchange. It is possible that the information given during PCH is provided to patients at wards with non-verbal handover as well, albeit without structure and not in the handover situation. Perhaps this is also the key to understanding why no subscales other than exchange of information were affected by PCH—nursing staff already inform patients, are available and provide respectful and compassionate care. The high levels of patient overall satisfaction and satisfaction with nurses also support this notion. These high levels might also have contributed to a ceiling effect, making it difficult to improve these variables, as they were already high at baseline.

Other studies using patient satisfaction as an outcome measure for bedside handover styles have found general increases after implementation [21, 29]. None have, to our knowledge, used the EORTC IN-PATSAT32 for evaluation and these studies were not performed in oncology settings. Therefore, our results cannot be related directly to other intervention studies. We can, however, compare the levels of EORTC IN-PATSAT32 mean scores to previous studies using the same instrument. Our results correspond well with the findings from Brédart et al. (2005), with the exception of comfort and cleanliness (COM), for which patients in the present study gave a lower score. In a study from the same department, preceding this one by a year, the patient satisfaction scores from EORTC IN-PATSAT32 correspond well with the results from both the baseline and intervention phases in the present study[30]. This implies that our results are stable over time and that the improvement in information exchange, as seen in the intervention ward, could be related to the introduction of PCH.

One strength of the study is the use of a validated instrument for measuring patient satisfaction. The EORTC IN-PATSAT32 was specifically developed for inpatient oncological care and has been proved valid and reliable [28]. This is also, to our knowledge, the first comparative study with a control group evaluating nurse handover styles in oncology care. As Cohen et al. (2010) state, there is a significant lack of well-designed studies investigating handovers, especially using patient-reported outcomes [31].

Overall, the response rate was relatively low (58% at T0 and 61% at T1) and could cause a sampling bias. Since a returned questionnaire was considered informed consent, we could not gather clinical variables on non-responders, as they had not formally given their consent for us to scrutinize their EHRs. Knowing that patients are seriously ill and vulnerable when receiving inpatient cancer care, we were very cautious when sending our reminders. If there was any indication that a patient was being admitted to another health care institution (most often a hospice or a rehabilitation unit), we refrained from sending a reminder, as we could not be certain which department they would then evaluate. This could, on the other hand, be positive, as we know for certain that the patients’ responses reflect the care of the studied wards and were not related to care given elsewhere.

There are limitations relating to the design of the study. We have only unpaired data as each patient could respond only once. Therefore, we have two independent groups and cannot compare results over time; rather, only at points of measurement. This, together with lack of randomization, opens up for uncontrolled confounders. It was not considered possible to randomize patients to different handover styles within the same ward because of the obvious risk of a spill over effect. In addition, it would not have been ethically defendable to randomize patients to the different wards upon admission because of the cancer site-specific specializations; for example, we could not allow a patient with head/neck cancer be treated at a gyno-oncological ward because of this study. Another option might have been cluster randomization where wards are randomized to either intervention or control. This is not a successful strategy when evaluating a complex intervention highly dependent on encouragement and the staff’s willingness to participate, as described by Malfait et al (25). We did, however, control various clinical variables that could determine patient satisfaction (6) and included a baseline measurement including all wards.

## Conclusion

Minor differences in patient satisfaction were found between the intervention ward and the control wards after implementing person-centred handover. The subscale related to the exchange of information between caregivers was improved in the intervention ward at the second point of measurement while no other changes in patient satisfaction were detected. PCH seemed to be feasible in an oncological inpatient setting and affected patient’s perceptions of information exchange between caregivers. Further evaluation of its impact on for example information exchange is needed.

The intervention presented in this study, PCH, can be of interest for all professionals in inpatient nursing. Researchers and nurse managers should be aware of how patient satisfaction could be affected by different handover styles. Patient satisfaction and efficient nurse handovers are core elements in quality care, and this study adds up to the current knowledge about their relation.

## Supporting information

S1 Appendix A supplementary Excel-spreadsheet containing all data analyzed and reported in the present study. Patient characteristics and results from the EORTC IN-PATSAT32 questionnaire (per item).
(XLSX)
